# Enhancing healthy eating and active environments in early learning settings: findings from the eHealth study

**DOI:** 10.3389/fnut.2026.1873291

**Published:** 2026-08-24

**Authors:** Lynne M. Z. Lafave, Joyce Hayek, Ceilidh McConnell, Bjorn Winkens

**Affiliations:** 1 CHEERS Lab, Department Health and Physical Education, Mount Royal University, Calgary, AB, Canada; 2 Faculty of Health, Medicine and Life Sciences, Care and Public Health Research Institute (CAPHRI), Department of Methodology and Statistics, Maastricht University, Maastricht, Netherlands

**Keywords:** early childhood education and care, eHealth intervention, interconnected model of professional growth, linear mixed models, nutrition, physical activity, professional development, social cognitive theory

## Abstract

**Introduction:**

Early childhood educators are important influencers of children’s eating and activity behaviors, making them key targets for interventions aimed at improving nutrition and physical activity practices and fostering healthier childcare environments.

**Objective:**

To evaluate the impact of the eHealth professional development program in improving early childhood education and care (ECEC) nutrition and physical activity environments, educators’ nutrition knowledge, attitudes, and behaviors, and educators’ perceptions of program acceptability.

**Method:**

This study was conducted in licensed ECEC centers in one Canadian province. The study used a two-armed quasi-experimental pre-test/post-test design with intervention and control groups and repeated measures to evaluate a 10-month eHealth program informed by Social Cognitive Theory and the Interconnected Model of Professional Growth Theory. The program aimed to enhance educators’ capacity to implement evidence-based nutrition and physical activity practices. Delivered in annual cohorts from 2019 to 2021, it comprised two phases: (1) a 12-week series of online educational modules with accompanying virtual Communities of Practice meetings, and (2) a five-month follow-up period involving monthly implementation-coaching virtual Communities of Practice meetings. Validated questionnaires assessed center-level and educator-level outcomes at baseline, five (midpoint), and 10 months (end-of-program). Semi-structured interviews explored perceptions of program acceptability.

**Results:**

Compared with controls, significant improvements in center-level nutrition and physical activity environments (mean change 0.362, *p* = 0.002, Cohen’s d = 0.72), and educator nutrition knowledge, attitudes, and behaviors (mean change 5.467, *p* = 0.023, Cohen’s d = 0.69) were observed at midpoint and maintained at the end-of-program. Qualitative findings indicated high acceptability, with educators describing the program as relevant, feasible, and supportive of integrating practices into daily routines.

**Conclusion:**

The eHealth professional development program was acceptable and associated with improvements in educators’ knowledge and adherence to nutrition and physical activity practice guidelines, with sustained effect over time. Scalable, digitally delivered professional development may offer a promising strategy to support healthier ECEC environments.

## 1 Introduction

Early childhood is a critical period where eating and physical activity habits are established, often persisting into adulthood (1, 2) that influence non-communicable disease risk (3). As childcare use has expanded (4), early childhood education and care (ECEC) settings have become a key context shaping children’s eating and movement behaviors.

International evidence identifies ECEC settings as promising venues for influencing health-promoting behaviors at an early age and underscores the importance of multi-component, multi-level intervention approaches (5). Given the considerable time ECEC educators spend with children, they are well placed to foster healthy behavior development (6). Large-scale international datasets highlight both the pivotal role of educator practices for children’s learning and well-being and the need for sustained professional development to strengthen ECEC quality (4), reinforcing calls to invest in educator capacity as a core strategy for improving children’s health behaviors in ECEC settings.

ECECs face challenges in applying both nutrition and physical activity guidance. Studies point to low compliance with dietary recommendations (7), limited access to healthy foods (8), educators’ reported need for support in mealtime practice skills (9), and applying the revised Canadian Food Guide in ECEC (10). Barriers such as limited educator knowledge, practical skills, and access to resources further impede guideline implementation (11). Despite evidence that ECEC settings implementing educator-led strategies are key to improving physical activity in young children (12, 13), implementation of physical activity guidelines remains challenging in ECEC, as many preschoolers spend the majority of their day in sedentary pursuits while in care (14). Evidence indicates that educators often feel underprepared to lead structured physical activity, citing gaps in knowledge and practical skills, as well as limited time and environmental support (15). Collectively, this highlights the need for comprehensive professional development to strengthen educators’ knowledge and adherence to optimal nutrition and physical activity practices as a child health priority.

eHealth interventions offer potentially cost-effective approaches to delivering such professional learning, with emerging evidence of feasibility, acceptability, and positive impacts on nutrition and physical activity practices in ECEC settings globally (16–19). A foundational example is the Nutrition and Physical Activity Self-Assessment for Child Care (NAP SACC) program, an established health intervention designed to promote healthy behaviors within early childhood education programs via in-person delivery (20). This model was later adapted into an online format (Go NAPSACC) and shown to be cost-effective in improving the nutritional environments of childcare services (19, 21). This framework has since been scaled internationally, serving as the benchmark for localized adaptations such as an Australian web-based intervention aimed at promoting healthy eating practices, which proved low-cost, highly acceptable to staff, and successful in improving the implementation of healthy eating policies (16). Despite these international successes, a recent scoping review revealed that few eHealth interventions have been developed specifically within the Canadian context, with existing domestic research primarily focusing on physical activity and active play (22–24). ECEC in Canada operates under decentralized, provincially regulated jurisdictions, which require alignment with distinct domestic benchmarks such as Canada’s Food Guide and the Canadian 24-Hour Movement Guidelines for the Early Years. Implementation science highlights that public health interventions do not seamlessly cross geopolitical borders, and transported interventions frequently suffer from poor engagement due to misalignment with local institutional structures (25). Consequently, a tailored Canadian eHealth intervention that aligns with regional regulatory standards and cultural contexts is fundamental to support professional learning program uptake in these settings.

To address this gap, we examined an eHealth professional development program designed to strengthen educators’ knowledge, skills, and capacity to implement evidence-informed nutrition and physical activity practices in ECEC settings. The primary research question was: Does participation in the eHealth professional development program improve ECEC nutrition and physical activity environments? This study also aimed to assess the program’s impact on educators’ nutrition-related knowledge, attitudes, and behaviors, and explore educators’ perceptions of the intervention. We hypothesized that participation in the program would lead to improvements in ECEC nutrition and physical activity environments and educator-level nutrition-related outcomes compared with practice-as-usual control.

## 2 Materials and methods

### 2.1 Study setting and design

This study formed part of a larger evaluation of the “CHEERS HEAPful of FUN: raising healthy Albertans” eHealth professional development program for early childhood educators, for which the study protocol has been published elsewhere (26). The present article is the primary outcomes paper, reporting quantitative center- and educator-level results from the quasi-experimental trial, along with educator perspectives on program experience; complementary in-depth qualitative analyses are reported separately. The broader evaluation included quantitative and qualitative assessments of implementation and center-, educator-, and child-level outcomes. A two-armed, quasi-experimental pre-post mixed-methods design with repeated measures was used to assess program impact in community-based ECEC settings. The design was selected to evaluate the intervention under routine service conditions, where centers participated in either the intervention or practice-as-usual control group to enable comparison between groups over time (27).

### 2.2 Participants and recruitment

ECEC centers, educators nested within, were identified from a provincial registry in one Canadian province and stratified by postal code to represent large, medium, and small population centers (28). Eligible centers were provincially licensed, provided care for at least 15 children aged 2–5 years, had internet and computer access, and were not participating in other nutrition or physical activity interventions. Unlicensed centers, family day homes, and after-school care programs were excluded. Center directors were contacted by telephone, provided study information, and given the option to be in the intervention or control group based on their center’s available time and capacity. Centers that agreed received email materials (instructions, consent forms, survey links) and nominated educators (typically two teachers and the director) to participate.

Power analysis using G*Power indicated 128 educators (64 per group) would provide 80% power at *α* = 0.05 to detect a medium effect (Cohen’s d = 0.5) on the primary outcome. To account for clustering within ECECs, the sample was adjusted using a design effect DE = 1 + (m-1) × ICC, with an average cluster size of 3 and ICC (0.0076) from pilot data, yielding DE = 1.0152. Planning for attrition (29), we aimed to recruit at least 186 educators (64 × 1.0152)/0.7 = 93 per group.

Recruitment occurred in annual cohorts beginning in 2019; this analysis includes participants from the 2019–2021 cohorts. Ethical approval was granted by the Human Research Ethics Board at Mount Royal University (no. 101768), and all participants provided digital informed consent.

### 2.3 Intervention

#### 2.3.1 Theory of change

The eHealth professional development program was informed by Social Cognitive Theory (SCT) and the Interconnected Model of Professional Growth (IMPG), each underpinning mechanisms for individual and collective change (30, 31). Drawing on SCT, the online modules and reflection activities targeted educators’ knowledge, outcome expectancies, and self-efficacy for providing healthy eating and activity environments; while modeling and practical examples were used to support observational learning in everyday practice. In line with IMPG, the virtual Communities of Practice (vCoP) sessions provided structured opportunities for reflection on practice, enactment of change through center-based ‘challenges’, and feedback within the personal and external domains, to nudge iterative changes in professional practice and ECEC environments and improved child health outcomes. Intervention components were structured to guide educators through cycles of reflection, experimentation, and refinement, consistent with SCT and IMPG assumptions that repeated practice in supportive social contexts strengthens self-efficacy and behavior change.

#### 2.3.2 Intervention procedure

The program was delivered over 10 months and comprised two phases of online professional development with both synchronous and asynchronous components.

In Phase 1 (12 weeks), educators completed weekly self-paced online learning modules (*n* = 12; approximately 1–2 h each), complemented by vCoP sessions (*n* = 12). Each vCoP group included approximately 5–8 educators from the same provincial cohort, organized by position (teacher or director), and met in 1-h videoconference sessions. Learning modules addressed core topics in nutrition and physical activity for young children, including eating patterns and the eating environment, physical literacy and activity, the role of sleep in health, and educator wellbeing. Content combined short instructional videos, practical resources, and structured reflection activities accessible by computer or mobile device. Module-related vCoP sessions were facilitated by early childhood education faculty and focused on discussing module content, sharing experiences, and reflecting on practice.

Phase 2 (5 months) comprised monthly, one-hour implementation-coaching vCoP meetings in which educators from the same center met together, facilitated by health promotion practitioners with expertise in nutrition, public health, or physical activity. These meetings aligned with the broad module concepts but used a flexible format that empowered educators to focus discussions on their needs with a view to applying learning to center routines, addressing barriers, and co-developing strategies for change.

In week 9, educators were invited to select one practice change (“challenge”) based on module content, implement it in their center, and write up the reflections to share in subsequent vCoP sessions.

#### 2.3.3 Control arm

Participants in the practice-as-usual control group did not have access to the program during the study period.

### 2.4 Outcome measures and data collection

A mixed-method approach was used to assess educator- and center-level outcomes, and the acceptability of the intervention. Quantitative data were collected at three time points for intervention group [baseline (T1), 5 months (T2), 10 months (T3)] and two time points for the control group [baseline (T1), 10 months (T3)] using online surveys. Surveys were administered via a secure web-based platform compatible with computers and mobile devices.

#### 2.4.1 Quantitative assessment

##### 2.4.1.1 Creating healthy eating and active environments survey

The primary outcome, ECEC nutrition and physical activity environments, was assessed using the creating healthy eating and active environments survey (CHEERS), a validated educator-completed audit tool designed for center-based programs. The tool includes 59 items across four subscales: foods served, healthy eating environment, program planning, and physical activity environment. Response options (7-point Likert) are averaged for a final score (range 1–7) where higher scores indicate closer alignment with best practice (32). Prior psychometric studies have established content validity, internal consistency (Cronbach’s *α* = 0.91), intra- and inter-rater reliability, and concurrent validity with expert dietitian assessment for this tool (32–34).

##### 2.4.1.2 Canadian behavior, attitude and nutrition knowledge survey

Educator nutrition-related change was assessed using the 60-item Canadian behavior, attitude, and nutrition knowledge survey (C-BANKS 2.0). The knowledge subscale is scored as correct/incorrect, and the attitude and behavior subscales use a 7-point Likert scale. The total score is determined by averaging the mean percent subscale scores (score range 14.30–100), with higher scores reflecting more favorable outcomes. C-BANKS 2.0 has demonstrated content validity, internal consistency (Cronbach’s *α* = 0.93), construct validity, and responsiveness for early childhood educators (35).

##### 2.4.1.3 Mindful eating questionnaire

Educator mindfulness in eating behavior was measured using the 28-item mindful eating questionnaire (MEQ) consisting of five subdomains (disinhibition, awareness, external cues, emotional response, and distraction) scored on a 4-point Likert scale, where higher scores signify more mindful eating and correlates with higher diet quality (36). Total score is calculated by averaging the subscales. The MEQ has demonstrated internal consistency (Cronbach’s *α* = 0.64), construct validity, responsiveness (37), and has been cross-culturally adapted and validated in multiple languages (38).

##### 2.4.1.4 Demographics

Demographic characteristics collected included educator age (years), gender, education, position (director/teacher/cook/owner), ECEC auspice (for-profit vs. not-for-profit), number of children per ECEC, and food provision (ECEC- vs. family-provided meals). Education levels, categorized according to provincial standards, are described as having completed a 45-h child development–related course (Level 1), completed a 1-year early learning and child care (ELCC) certificate (Level 2), completed a 2-year ELCC diploma (Level 3), and completed a 4-year full-time post-secondary credential in a related or equivalent ELCC field (University degree).

##### 2.4.1.5 Implementation fidelity

Implementation fidelity for each educator was monitored using learning platform analytics (modules completed) and vCoP attendance logs. Fidelity was calculated as dose received (modules and sessions attended) divided by dose delivered (12 modules and 12 sessions offered), with at least 80% of planned dose classified as high fidelity, consistent implementation fidelity guidance (39).

#### 2.4.2 Qualitative assessment

At the end-of-program, educators in the intervention arm were invited to take part in semi-structured interviews exploring the acceptability, perceived impact, and implementation of the program (Supplementary material S1). An interview guide focused on experiences with the online modules, vCoP sessions, delivery format, and perceived barriers and facilitators. Interviews were conducted online, audio-recorded with consent, and transcribed with identifying information removed to support anonymity in analysis.

### 2.5 Data analysis

#### 2.5.1 Statistical analysis

Data were analyzed using IBM SPSS Statistics for Windows version 28 (Armonk, NY: IBM Corp), with statistical significance was set at *p* ≤ 0.05. Descriptive analysis included means and standard deviations for continuous variables, count (%) for categorical variables. Independent-samples t-tests or chi-square tests compared baseline demographics between groups. Fisher’s exact test or Fisher–Freeman–Halton exact test were used when at least one of the expected frequencies were less than 5, with two-sided *p*-value computed as 2*one-sided p-value.

Linear mixed model (LMM) analyses were used to analyze the intervention effects on educators’ CHEERS, MEQ and C-BANKS 2.0 scores. As measurements were repeated within educators and educators nested within ECECs, a three-level model with repeated measurements as first level, educators as second level, and ECECs as third level. Fixed effects included group (Intervention-Control), time (T1, T3), and their interaction (group*time) to assess intervention effects at T3 after baseline differences correction (T1). To correct for cohort effect, the variable cohort (using dummy variables) and its interaction (time*group*cohort, time*cohort, group*cohort) were added to account for possible difference in cohort-dependent intervention effects. Non-statistically significant interaction terms were removed in a top-down procedure. Analyses were adjusted for demographic covariates: age, auspice, education, gender, position, number of children per center, and food provision. A random intercept on ECEC center level accounted for clustering (correlation between educators within same center), while a random slope to account for heterogeneity of center level variance over time was not included as it could not be estimated. An unstructured covariance matrix was used to account for correlation between repeated measures within an educator.

Using the same LMM approach, but only including time (T1, T2, T3) as a fixed factor, separate analyses examined the time effect within the intervention group, with pairwise comparisons between the time points (T2 vs. T1, T3 vs. T1, T3 vs. T2).

As sensitivity analyses, all analyses were also applied to the group of participants who had complete data, i.e., complete case analysis (CCA). This CCA showed similar results and same conclusions, so only the results of the main analyses are reported.

Standardized effect sizes (Cohen’s d) were calculated by dividing the estimated treatment effect (difference in corrected mean change scores between groups) by the pooled SD of change scores using the residual variance and covariance of the LMM. Within-group effects were computed as the mean change score divided by the change score SD.

The effect estimates were reported with 95% confidence intervals, *p*-values.

#### 2.5.2 Qualitative analysis

Interview transcripts were analyzed using Braun and Clarke’s six-step thematic analysis approach (54). The third author first familiarized themself with the data and generated initial codes across the dataset. The first and third authors then iteratively reviewed and refined codes into candidate themes, which were checked against the data, defined, and named, with illustrative extracts selected to represent each theme.

## 3 Results

### 3.1 Participant selection and characteristics

**Figure 1:**
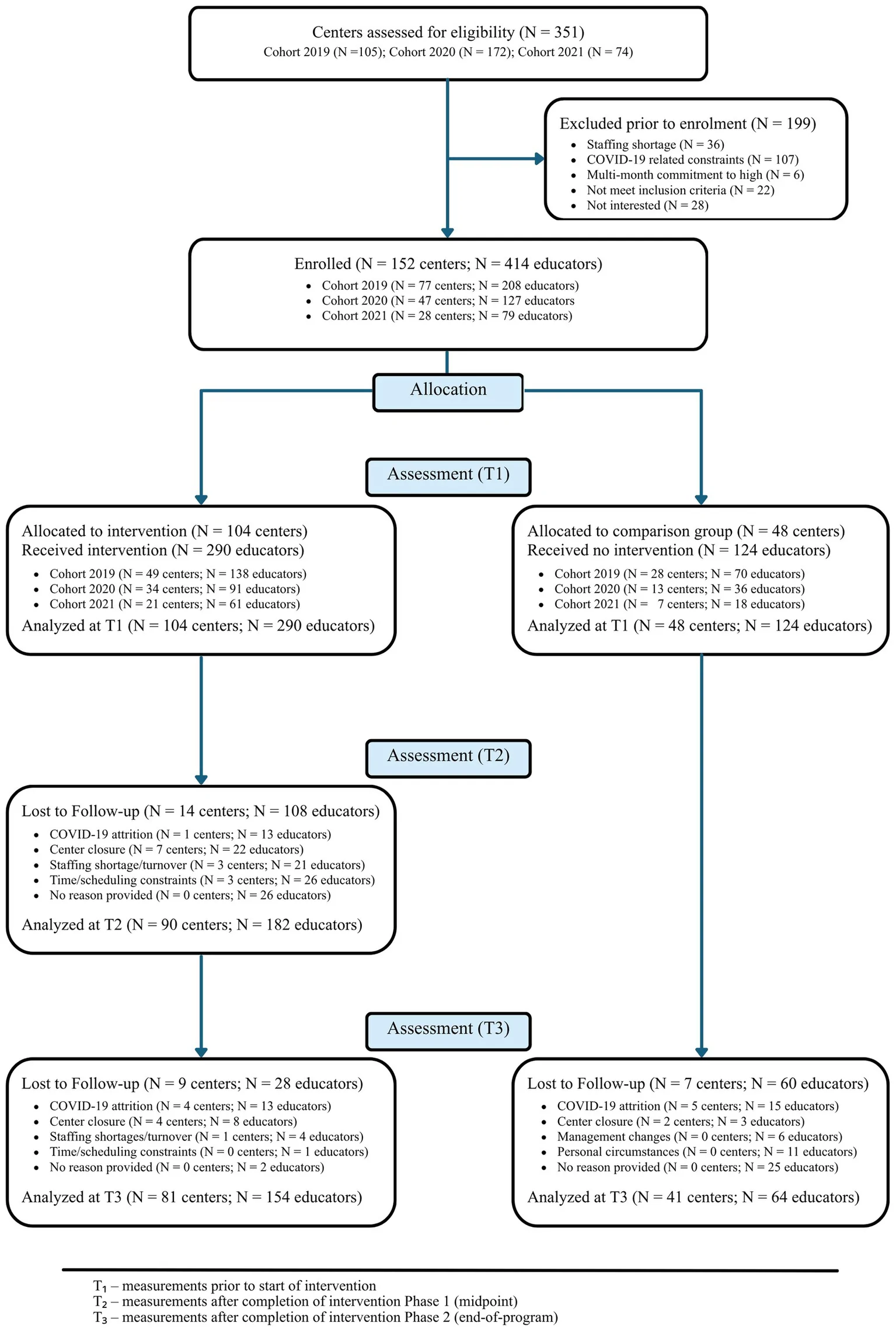
Participant flow diagram outling the recruitment, allocation, follow-up, and final analysis of centers and educators.

The recruitment, allocation, and tracking of participants through the sequential phases of the study are visually detailed in the participant flow diagram (Figure 1). Across all cohorts, 414 educators (290 intervention, 124 control) from 152 centers (104 intervention, 48 control) were enrolled; post-intervention outcome data was available for 218 (53%) educators (154 intervention, 64 control). Overall, 196 (47%) educators were lost to follow-up, with attrition distributed similarly between the intervention (136/290; 47%) and control (60/124; 48%) groups. According to educators’ reports at withdrawal, dropout was primarily attributed to pandemic-related disruptions, childcare center closures, staff shortages, ECEC management changes, time constraints, and personal circumstances. Among the subsample of intervention educators who completed the study and provided post-intervention data (n = 154), 95% achieved at least 80% of the intended professional development dose, indicating high exposure to both asynchronous and synchronous components of the program.

The demographic, professional, and operational characteristics of the participating educators and childcare centers collected at baseline are summarized in Table 1. Across cohorts, were generally similar at baseline; with differences observed in age in 2019 and food provision, education level, and age in 2020.

**Table 1:** Baseline participant demographics by cohort.

Characteristics	Cohort 2019		Cohort 2020		Cohort 2021	
	IV	CTRL	IV	CTRL	IV	CTRL
Educators, *n*	138	70	91	36	61	18
Age in years, mean ± SD	41.5 ± 11.9^**a**^	37.2 ± 10.8	38.9 ± 11.0	44.1 ± 11.7^**a**^	39.9 ± 10.7	37.8 ± 10.2
Gender, *n* (%)						
Women	131 (94.9)	69 (100)	89 (97.8)	36 (100)	59 (96.7)	17 (94.4)
Men	7 (5.1)	1 (0)	2 (2.2)	0 (0)	2 (3.3)	1 (5.6)
Education level, *n* (%)						
Level 1	23 (16.7)	10 (14.3)	15 (16.5)	1 (2.8)^**b**^	11 (18.0)	3 (16.7)
Level 2	18 (13.0)	10 (14.3)	14 (15.7)	4 (11.1)	5 (8.2)	0 (0)
Level 3	66 (47.8)	26 (37.1)	47 (50.6)	18 (50.0)	30 (49.2)	12 (66.6)
University degree	31 (22.5)	24 (34.3)	15 (16.9)	13 (36.1)	15 (24.6)	3 (16.7)
Educator position, *n* (%)						
Director	39 (28.3)	24 (34.3)	16 (17.6)	13 (36.1)	19 (31.1)	6 (33.3)
Teacher	89 (64.5)	40 (57.1)	63 (69.2)	22 (61.1)	35 (57.4)	12 (66.7)
Cook	2 (1.4)	1 (1.4)	2 (2.2)	0 (0)	3 (4.9)	0 (0)
Operator	8 (5.8)	5 (7.1)	10 (11.0)	1 (2.8)	4 (6.6)	0 (0)
Auspice, *n* (%)						
Children per center, mean ± SD	47.4 ± 37.2	44.2 ± 29.9	35.2 ± 27.5	38.4 ± 22.3	29.9 ± 23.6	22.4 ± 9.5
Non-profit	40 (29.0)	21 (30)	36 (39.6)	12 (33.3)	23 (37.7)	8 (44.4)
For-profit	98 (71.0)	49 (70)	55 (60.4)	24 (66.7)	38 (62.3)	10 (55.6)
Food provision, *n* (%)						
Center-provided	123 (89.1)	67 (95.7)	65 (71.4)	33 (91.7)^**b**^	56 (91.8)	14 (77.8)
Parent-provided	15 (10.9)	3 (4.3)	26 (28.6)	3 (8.3)	5 (8.2)	4 (22.2)

Analysis comparing intervention with control within a cohort: ^a^*p* < 0.05 for Independent Samples *t*-test, ^b^*p* < 0.05 for chi-square test; n, sample size; IV, Intervention group; CTRL, Practice-as-usual control group; SD, Standard Deviation.

### 3.2 Intervention effects

The intervention produced moderate-to-large effects in center-based nutrition and physical activity environments and in educators’ nutrition-related knowledge, attitudes, and behaviors compared with the practice-as-usual control group (Table 2). After adjusting for baseline scores, cohort, and educator/center characteristics (gender, age, auspice, education, food provision, number of enrolled children), the intervention group showed a significantly greater increase in CHEERS scores (mean change 0.362, *p* = 0.002; Cohen’s d = 0.72) and C-BANKS 2.0 scores (mean change 5.467, *p* = 0.023; Cohen’s d = 0.69) from baseline to end-of-program than the control group. No statistically significant between-group differences were found for MEQ scores, suggesting limited change in educators’ mindful eating behaviors.

**Table 2:** Observed means (SD) and estimated intervention effects on CHEERS, MEQ, and C-BANKS 2.0 scores between intervention (IV) and practice-as-usual control groups (CTRL) across cohorts.

Outcome	Group	Baseline (T1)		End-of-program (T3)		Intervention effect^a^	
		*N*	Mean (SD)	*N*	Mean (SD)	Difference in mean change (95%CI)	*p*-value
CHEERS	IV	290	5.468 (0.831)	154	5.886 (0.762)	0.362 (0.138; 0.586)	0.002
	CTRL	124	5.695 (0.685)	64	5.825 (0.609)		
MEQ	IV	237	2.957 (0.302)	143	3.029 (0.349)	0.003 (−0.159; 0.164)	0.973
	CTRL	91	2.915 (0.317)	58	2.947 (0.304)		
C-BANKS 2.0^†^	IV	194	68.15 (10.77)	99	74.29 (10.47)	5.467 (0.77; 10.16)	0.023
	CTRL	88	69.61 (11.38)	46	71.03 (11.75)		

^a^Corrected for gender, age, auspice, education level, food provision, number of children enrolled in ECEC center, and cohort. ^†^Only data from the 2019 and 2020 cohort were used for the C-BANKS 2.0 analysis. IV, Intervention group; CTRL, Practice-as-usual control group; SD, Standard Deviation; CI, Confidence Interval; CHEERS, Creating Healthy Eating and active Environments survey; MEQ, Mindful Eating Questionnaire; C-BANKS, Canadian Behavior, Attitude and Nutrition Knowledge Survey.

Within the intervention group (Table 3), CHEERS scores increased significantly both from baseline to midpoint (*p* < 0.001; Cohen’s d = 0.69) and to end-of-program (p < 0.001; Cohen’s d = 0.82), reflecting large, sustained improvements in center-based nutrition and physical activity environments. C-BANKS 2.0 scores also rose significantly from baseline to midpoint (p < 0.001; Cohen’s d = 0.43) and to end-of-program (p < 0.001; Cohen’s d = 0.70), reflecting moderate-to-large effects in educators’ nutrition knowledge, attitudes, and behaviors over time. In contrast, changes in MEQ scores were small and not statistically significant.

**Table 3:** Observed means (SD) and estimated changes in CHEERS, MEQ and C-BANKS 2.0 for the intervention group.

Observed Mean ± SD, N	Timepoint comparison	Mean change	95% CI	*p*-value
CHEERS				
T1 (*N* = 290, 5.468 ± 0.831)	T2-T1	0.406	0.324; 0.487	<0.001
T2 (*N* = 182, 5.873 ± 0.721)	T3-T2	0.067	−0.013; 0.147	0.100
T3 (*N* = 154, 5.886 ± 0.762)	T3-T1	0.473	0.387; 0.558	<0.001
MEQ				
T1 (*N* = 237, 2.975 ± 0.302)	T2-T1	0.018	−0.019; 0.055	0.342
T2 (*N* = 175, 3.017 ± 0.330)	T3-T2	0.014	−0.025; 0.053	0.485
T3 (*N* = 143, 3.029 ± 0.349)	T3-T1	0.032	−0.012; 0.075	0.152
C-BANKS 2.0				
T1 (*N* = 194, 68.15 ± 10.77)	T2-T1	5.481	2.525; 8.438	<0.001
T2 (*N* = 69, 74.99 ± 13.66)	T3-T2	0.428	−2.628; 3.484	0.781
T3 (*N* = 99, 74.29 ± 10.47)	T3-T1	5.910	4.278; 7.541	<0.001

SD, Standard Deviation; N, sample size; CI, Confidence interval; T1, Baseline; T2, Midpoint; T3, End-of-program. CHEERS: Creating Healthy Eating and active Environments survey, MEQ: Mindful Eating Questionnaire, C-BANKS: Canadian Behavior, Attitude and Nutrition Knowledge Survey. Significant difference *p* ≤ 0.05.

#### 3.2.1 Program acceptability

Across the three cohorts, 64 educators completed interviews; most were women (*n* = 63) with an average age of 41.5 years. Directors (*n* = 34) and teachers (*n* = 30) were similarly represented, with predominantly Level 3 certification (*n* = 37) or university degree (*n* = 19). Center auspice (non-profit, for-profit) was similarly represented (*n* = 38, *n* = 26) with food provision primarily provided by the center (*n* = 55). These characteristics largely mirror the demographics of the overall participant group.

Educators expressed consistently high levels of satisfaction with the program, emphasizing its acceptability and alignment with their professional needs.

The digital format was repeatedly described as a strong feature of the program, offering both accessibility and convenience. Educators highlighted clear organization, flexible pacing, and ease of access as key strengths that supported engagement. One teacher noted appreciating being able to *“chip away at it at your own pace”* within each week (Teacher 45).

vCoP sessions were frequently described as particularly valuable for deepening learning and reflection, as educators could discuss shared challenges and explore practical strategies with peers. As one participant explained, these discussions “*really made me think more critically about, okay, what are we actually doing with our day? What changes can we make and how do we make them.”* (Teacher 10).

Educators emphasized the practicality of program materials, describing them as directly applicable to daily practice and helpful for communicating with families and colleagues. Having ready-to-use tools and reference materials was seen as increasing confidence to implement nutrition and physical activity practices: “*they were a great source for knowledge and for … physical activity, nutrition, planning and everything”* (Teacher 43).

Time was the main barrier in participation, with some educators finding it challenging to dedicate uninterrupted time for modules or evening sessions along family responsibilities; several described the program as “time-consuming” despite its perceived value (Director 24; Teacher 12).

Nevertheless, a predominant theme was the value and importance educators believed the program enhanced their ECEC environment stating that *“in multiple staff meetings about how we would love for the rest of the [Center] to participate in it”* (Director 59) and for their professional field overall *“it would be great if most educators were brought to this program”* (Teacher 12).

## 4 Discussion

Our findings indicate moderate-to-large improvements in center-level nutrition and physical activity environments and in educators’ nutrition-related knowledge, attitudes, and behaviors associated with participation in the eHealth professional development program. These magnitudes suggest that the intervention yielded significant enhancements in educators’ capabilities and center practices. Improvements in center-level nutrition and physical activity environments were further supported by educators’ qualitative interview data, which described concrete changes in food provision, mealtime practices, and opportunities for active play within early learning and care settings.

### 4.1 Interpretation of main findings

The positive effects on educators’ self-reported nutrition and physical activity practices are consistent with prior work showing that online or web-based professional development can improve early year’s nutrition and physical activity environments and provider knowledge (40–42). Our results add to emerging evidence that eHealth professional development interventions can enhance adherence to best practice guidelines, demonstrating that digitally delivered programs can produce meaningful improvements in ECEC nutrition and physical activity environments.

The observed improvements in educators’ nutrition-related knowledge, attitudes, and behaviors are important given evidence that educators’ dietary behaviors shape mealtime environments and children’s eating behaviors (43, 44). This aligns with SCT and the IMPG, which emphasize the influence of personal and contextual factors on children’s behaviors through role modeling and iterative cycles of reflection and practice (30, 31). Targeting educators’ personal nutrition-related factors may be a critical lever for improving ECEC nutrition environments and ultimately children’s dietary habits, underscoring the value of educator-focused health education interventions.

Across both center-level (CHEERS) and educator-level (C-BANKS 2.0) outcomes, the largest gains occurred during the initial intensive phase that combined self-paced modules with vCoP (T2), with improvements largely maintained but not further increased during the later maintenance phase (T3). This pattern suggests that concentrated professional development and collaborative reflection may be particularly potent in driving change, whereas ongoing implementation support may help sustain rather than amplify these effects over time.

In contrast, changes in mindful eating (MEQ) were small and not statistically significant between or within groups. Mindful eating was addressed in one module, which may have provided insufficient intensity or repetition to shift established eating habits. Previous work suggests that cultivating mindful habits requires extended practice and exposure to support emotional awareness and self-regulation (45, 46). Although this intervention did not produce measurable changes in mindful eating, future integration of additional mindful eating content may be important, given that educators act as role models and can influence children’s eating behaviors (47).

### 4.2 Educator perspectives

Qualitative findings indicated that educators perceived the program as relevant, feasible, and well aligned with their professional roles and daily routines in ECEC settings. Structured opportunities for reflection within modules and CoP conversations were described as particularly helpful for translating new knowledge into practice, resonating with the professional growth model where enaction and reflection mediate changes in beliefs and classroom practices (31).

Educators emphasized the value of high-quality, flexible digital content in the program, reporting that clear organization, self-paced access, and directly applicable resources supported engagement despite competing demands on their time. These observations align with prior work showing that content quality and practical applicability drive educators’ uptake of digital professional development (48, 49). However, time constraints and scheduling challenges emerged as key barriers, highlighting the importance of designing eHealth professional development that can be integrated into educators’ workloads and organizational structures.

Consistent with these qualitative accounts, among the subsample of educators who completed post-test measures, a high proportion of educators completing at least 80% of the intended professional development dose (module and vCoP). These fidelity metrics, alongside qualitative reports of high perceived relevance, suggest that the intervention program was feasible and acceptable in routine ECEC settings and increase confidence that the observed effects reflect the program rather than limited uptake.

### 4.3 Limitations and strengths

This study should be interpreted considering some limitations. The quasi-experimental design of our study can be viewed as a limitation, however, allocating centers based on willingness and capacity provides a pragmatic view of implementation. All outcomes were self-reported, introducing the possibility of respondent bias. Planned objective assessments of center environments using observational tools were constrained by pandemic-related restrictions, limiting observational data for some cohorts and reducing opportunities to triangulate self-reported changes. Additionally, while child-level behavioral and anthropometric health metrics (e.g., physical activity levels) were captured as secondary outcomes within the broader trial registry as detailed in our published protocol (26), the primary aim of this specific evaluation focused on the physical childcare environment and educator-led practices. In complex public health and implementation science frameworks, modifying organizational structures and building educator capacity are recognized as foundational primary steps; structural changes to the environment are precursors required to catalyze long-term, sustainable shifts in individual child-level health behaviors. The study was conducted within a single regional early childhood education system, consequently, generalizability to other Canadian provinces or international settings with distinct, centralized educational governance and funding structures should be approached with caution. Nevertheless, while the specific policy content must be tailored to regional benchmarks, the fundamental architecture, feasibility, and delivery mechanisms of this eHealth professional development framework remain highly scalable and generalizable across diverse ECEC jurisdictions. Most participants were women, reflecting the Canadian ECEC workforce where women (96%) constitute the vast majority (50). Attrition occurred over the study period driven by pandemic-related disruptions. This dropout rate aligns closely with macro-level workforce trends from the same time period (51), where national reporting indicated that 59% of Canadian ECECs experienced staff departures (52), alongside broader international turnover spikes (53). Importantly, rates were similar between intervention and control groups, reducing the likelihood that differential dropout explains the observed between-group effects. Finally, the longitudinal timeline of this study spanned different phases of the COVID-19 pandemic, meaning some baseline data were captured prior to restrictions, while subsequent waves occurred under modified childcare operating procedures (e.g., altered meal service and physical activity spacing policies). While these shifting public health mandates introduced unavoidable environmental variability independent of the intervention, the inclusion of a concurrent practice-as-usual control group served to isolate and control for these macro-level secular trends. Furthermore, sensitivity analyses evaluating longitudinal wave and cohort effects demonstrated that overall intervention directions and significance remained stable across waves, suggesting the changing pandemic context did not fundamentally alter the primary conclusions.

Despite these limitations, this study has notable strengths. Few evaluations have used a multi-level, multi-component eHealth approach to target both nutrition and physical activity in Canadian ECEC settings, and this work is among the first to do so under routine service conditions. The mixed-methods design provided an integrated view of impact and acceptability, including how the program was experienced and implemented in practice. Among educators who completed the study, implementation fidelity was high, indicating strong engagement with the program. In addition, the use of linear mixed models allowed inclusion of all available data, accounted for repeated measures and clustering of educators within centers, and, provided estimates that are robust under a missing-at-random assumption. To further ensure the integrity of our longitudinal data, Complete Case Analyses (CCA) were performed as sensitivity analyses and these yielded the same conclusions as the main analyses, confirming that participant attrition did not skew the primary outcomes. The pragmatic delivery of the eHealth professional development program under routine service conditions enhances the applicability of findings to ECEC practice and policy.

## 5 Conclusion

The eHealth community-based professional development program appeared to improve early childhood educators’ knowledge, beliefs, and adherence to best nutrition and physical activity practices, with effects sustained over time. Educators perceived the program as highly valuable and practical within their work contexts, indicating good acceptability and feasibility in routine ECEC settings. Taken together, these findings suggest that scalable, digitally delivered professional development has considerable potential to support the creation of healthier ECEC environments and could be integrated into existing professional development and quality improvement systems to promote sustainable implementation. Future research should examine the scalability of this program across diverse jurisdictions and integrate it with system-level policies to sustain changes in practice over time.

## Data Availability

The datasets generated during and/or analyzed during the current study are not publicly available due to participants’ privacy under the REB approval. Requests to access the datasets should be directed to llafave@mtroyal.ca.
