# Supplementary material for: Enhancing healthy eating and active environments in early learning settings: findings from the eHealth study
Source: Front Nutr. 2026 Aug 24;13:1873291. doi: 10.3389/fnut.2026.1873291 (PMC13548801; doi:10.3389/fnut.2026.1873291)
Supplement: Supplementary file 1 [file Table_1.DOCX]

Supplementary Material

**Semi-Structured Interview Guide**

**(Select Questions)**

These last questions are about your thoughts and experience with the program.

a) What was your experience with the online modules and community sessions? (Including anything you found to be of value or anything you would change)
b) What was your experience with the Health Promotion Leader meetings? (Including anything you found to be of value or anything you would change)
c) What part of the program went well for you?
d) What suggestions do you have for the future?
e) Was there anything else that stood out to you?
